# Effect of urolithin A on intracellular survival of *Mycobacterium tuberculosis* by regulating AKT-FOXO1-mediated autophagy

**DOI:** 10.1128/msphere.00061-25

**Published:** 2025-04-10

**Authors:** Jing Bi, Li Song, Qinglong Guo, Xi Chen, Yaqi Gong, Haojia Wu, Fan Zhang, Jingbin Wang, Guoliang Zhang

**Affiliations:** 1 National Clinical Research Center for Infectious Diseases, Shenzhen Third People’s Hospital, Southwest Medical University, Shenzhen, China; 2 School of Public Health, Guangdong Medical University, Dongguan, China; 3 School of Medicine, Southern University of Science and Technology 255310, Shenzhen, China; 4 Shenzhen Hospital of Guangzhou University of Chinese Medicine (Futian), Shenzhen, China; Washington University in St. Louis School of Medicine, St. Louis, Missouri, USA

**Keywords:** *Mycobacterium tuberculosis*, urolithin A, HDT, autophagy, AKT-FOXO1 signaling pathway

## Abstract

Tuberculosis (TB), resulting from *Mycobacterium tuberculosis* (Mtb), is one of the leading causes of morbidity and mortality in humans worldwide. Host-directed therapy (HDT) is a novel approach for treating TB, particularly those with drug resistance. Urolithin A (UroA) produced through bioconversion of plant-derived ellagic acid by gut microbes has been proven to have multiple beneficial effects in a variety of diseases without showing undesired adverse reactions. However, whether UroA has antimycobacterial effect and the underlying mechanism has not yet been reported. Here, we found that UroA significantly inhibited Mtb growth within both macrophages and mice. Moreover, UroA promoted the activation of autophagy in Mtb-infected macrophages via the protein kinase B–Forkhead box protein O1 signaling pathway, which contributed to the antimycobacterial effect of UroA. Additionally, UroA suppressed the survival of clinically isoniazid (INH)-resistant Mtb (C2) within macrophages, and the combination of UroA and INH synergistically enhanced host elimination of Mtb H37Rv. Therefore, UroA may be utilized as a potential candidate for HDT and as an adjunctive therapy with first-line anti-TB drugs.

**IMPORTANCE:**

Host-directed therapy (HDT) is a novel approach for treating tuberculosis (TB), particularly those with drug resistance. Urolithin A (UroA) produced through bioconversion of plant-derived ellagic acid by gut microbes has been proven to have multiple beneficial effects in a variety of diseases without showing undesired adverse reactions. We found that UroA significantly inhibited *Mycobacterium tuberculosis* (Mtb) growth within macrophages. Moreover, UroA suppressed the survival of clinically isoniazid (INH)-resistant Mtb (C2) within macrophages, and the combination of UroA and INH synergistically enhanced host elimination of Mtb H37Rv. Therefore, UroA may be utilized as a potential candidate for HDT and as an adjunctive therapy with first-line anti-TB drugs.

## INTRODUCTION

Tuberculosis (TB), resulting from *Mycobacterium tuberculosis* (Mtb), is one of the leading causes of morbidity and mortality in humans. More than 10 million people were affected by TB, and an estimated 1.3 million deaths by TB have been reported in 2022 (1). The increasing prevalence of drug-resistant TB, such as rifampicin (the most effective first-line drug)-resistant TB, poses a severe threat to public health; therefore, the development of effective anti-TB drugs is vital (1).

Host-directed therapy (HDT) is a novel strategy for treating TB, particularly drug-resistant TB, by directly regulating the anti-TB activity of the host using small molecules. Mechanistically, HDT drugs improve mycobactericidal effects by modulating innate and adaptive immunity, promoting cell death, and relieving immoderate inflammation and tissue injury on the host. Recent studies have centralized repurposing Food and Drug Administration (FDA)-approved drugs for use in HDT against Mtb infections. Imatinib, which is used for treating chronic myeloid leukemia, facilitates Mtb clearance by improving the phagosomal acidification of human macrophages (2). Prednisone and dexamethasone alleviate Mtb-induced immunopathology in patients with TB (3). Increased degranulation of CD8+ T cells and proportion of specific interferon gamma-producing lymphocytes against Mtb has been observed upon blocking programmed cell death 1 (4). Alisporivir and desipramine synergistically suppress reactive oxygen species-mediated necroptosis and mitigate tissue damage in a zebrafish model (5). These studies suggest that repurposing FDA-approved drugs for use in HDT is a potentially effective strategy for anti-TB therapy.

Autophagy is a basic process, which sustains cellular homeostasis by targeting redundant or abnormal organelles and proteins involved in lysosomal degradation and recycling. Autophagy is an innate immune defense mechanism that plays a crucial part in host elimination of Mtb (6). Several HDT drugs can trigger host autophagy and exert mycobactericidal effects. Rapamycin promotes autophagy and Mtb clearance by suppressing the mammalian target of rapamycin complex 1 (7). Resveratrol-induced autophagy and phagosome–lysosome fusion promote the effective restriction of intracellular Mtb (8, 9). Anti-TB drugs, including isoniazid (INH), pyrazinamide, and bedaquiline, promote Mtb clearance by enhancing host autophagy irrespective of their direct antimycobacterial effects (10, 11). The active form of cathelicidin induced by vitamin D activates autophagic flux, which is critical for inhibiting intracellular Mtb growth (12). Therefore, triggering host autophagy using HDT drugs is an effective therapeutic strategy against Mtb infections.

Ellagic acid is a natural polyphenol and exists in plant-based foods and medicinal herbs. Ellagic acid is converted into urolithin A (UroA) by gut microbes (13). Many reports have shown that UroA ameliorates oxidation and inflammation in various tissues and has beneficial effects against tumors, aging, obesity, diabetes, and neurodegeneration, without showing undesirable adverse reactions (13–17). UroA promotes mitophagy, a type of selective autophagy, which contributes to the maintenance of mitochondrial homeostasis, counteracts age-associated decline in organ function (16), and improves antitumor immunity by inducing T memory stem cell formation (17), obesity-induced metabolic cardiomyopathy (14), and muscular dystrophy (18). UroA activates autophagy and reduces ischemic neuronal death by suppressing endoplasmic reticulum (ER) stress (19). Besides, UroA-mediated autophagy inhibits the proliferation, migration, and invasion of multiple malignancies (15, 17, 20) and exerts antidiabetic effects (21–24). These studies suggest that UroA-mediated autophagy has important beneficial effects in multiple diseases.

The UroA plays a critical part in the host fighting infections. In pediatric pneumonia, UroA relieves inflammation, oxidative stress, and ER stress by triggering autophagy (25). UroA inhibits the replication of Enterovirus 71 in infected cells by enhancing autophagy and apoptosis (26). Additionally, UroA alleviates *Helicobacter pylori*-induced inflammation and tissue injuries (27). However, whether UroA has a mycobactericidal effect and its underlying mechanisms remain unclear.

Therefore, this study aimed to evaluate the effect of UroA on Mtb growth and induction of autophagy in macrophages. The mechanism underlying UroA-induced autophagy activation in Mtb-infected macrophages and subsequent antimycobacterial effects were assessed. Additionally, the potential of UroA to suppress the survival of a clinically INH-resistant Mtb (C2) within macrophages and the combined effect of UroA and INH in inducing host elimination of Mtb H37Rv were analyzed.

## RESULTS

### UroA inhibits Mtb growth within macrophages

**Fig 1:**
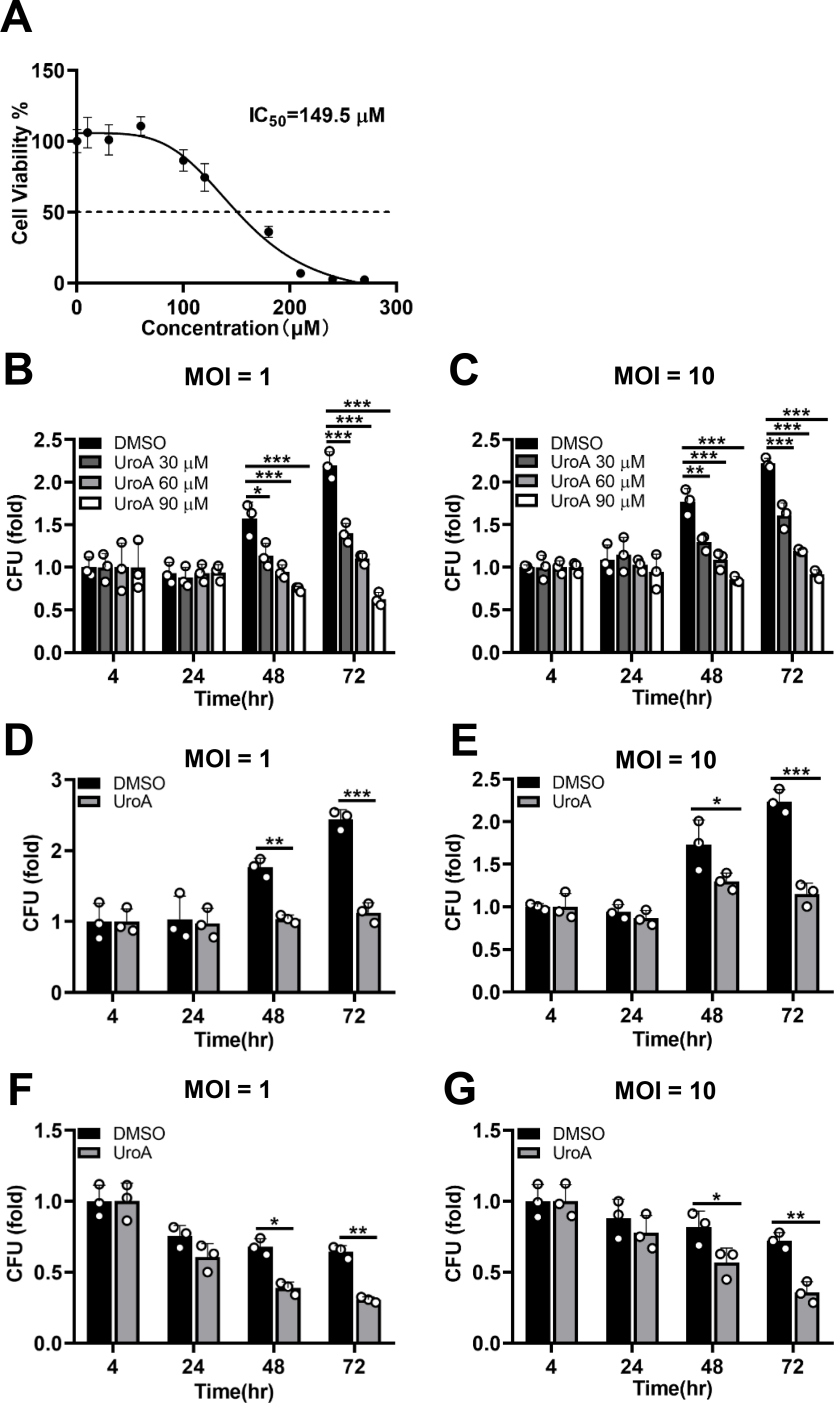
UroA inhibits Mtb growth within macrophages. (**A**) Cell viability of Mtb-infected THP-1 cells treated with UroA. (**B and C**) THP-1 cells were preincubated with UroA (30, 60, and 90 µM) for 1 h and then infected with Mtb H37Rv (MOI, 10:1 or 1:1) for 4 h. Cells were washed thrice with warm phosphate-buffered saline and then treated with UroA for another 24, 48, and 72 h. The number of CFUs was determined. (D–G) THP-1 cells (**D and E**) and BMDMs (**F and G**) pretreated with UroA (60 µM) were infected with Mtb H37Rv (MOI, 10:1 or 1:1) for 4 h. The infected cells were washed and stimulated as described in panel **B**. The number of CFUs was determined. The data are presented as mean ± SD of three independent experiments. Unless indicated otherwise, there is no significant difference; *P < 0.05; **P < 0.01; ***P < 0.001.

UroA plays a critical part in the host fighting infections (25–27). Whether UroA has a mycobactericidal effect remains unclear. Firstly, cell viability assay was performed in Mtb-infected THP-1 cells at 72 h in the presence or absence of UroA. As shown in Fig. 1A, the 50% inhibitory concentration of UroA was 149.5 µM. In the present study, we selected ≤90 µM of UroA for follow-up study as the cell viability of THP-1 cells was approximately 100% (Fig. 1A). Next, we performed colony-forming units (CFUs) assay to assess the antimycobacterial efficacy of UroA. UroA significantly reduced the intracellular survival of Mtb in THP-1 cells in a dose-dependent manner either at a multiplicity of infection (MOI) = 1 or 10 (Fig. 1B and C). Similarly, the intracellular Mtb growth was significantly inhibited in UroA-treated THP-1 cells (Fig. 1D and E) and bone marrow-derived macrophages (BMDMs; Fig. 1F and G) at a concentration of 60 µM. We hypothesized that the antimycobacterial activity of UroA was owing to its direct inhibition of Mtb. However, our data demonstrated that the minimum inhibitory concentration of UroA was >1.2 mM (Table 1). Together, these data indicate that UroA enhances host elimination of Mtb, probably by enhancing macrophage function.

**TABLE 1:** MIC of Mtb

Drug	MIC (mg/L)
UroA	>273.84 (1.2 mM)
INH	0.02

### UroA enhances autophagy flux within Mtb-infected macrophages

**Fig 2:**
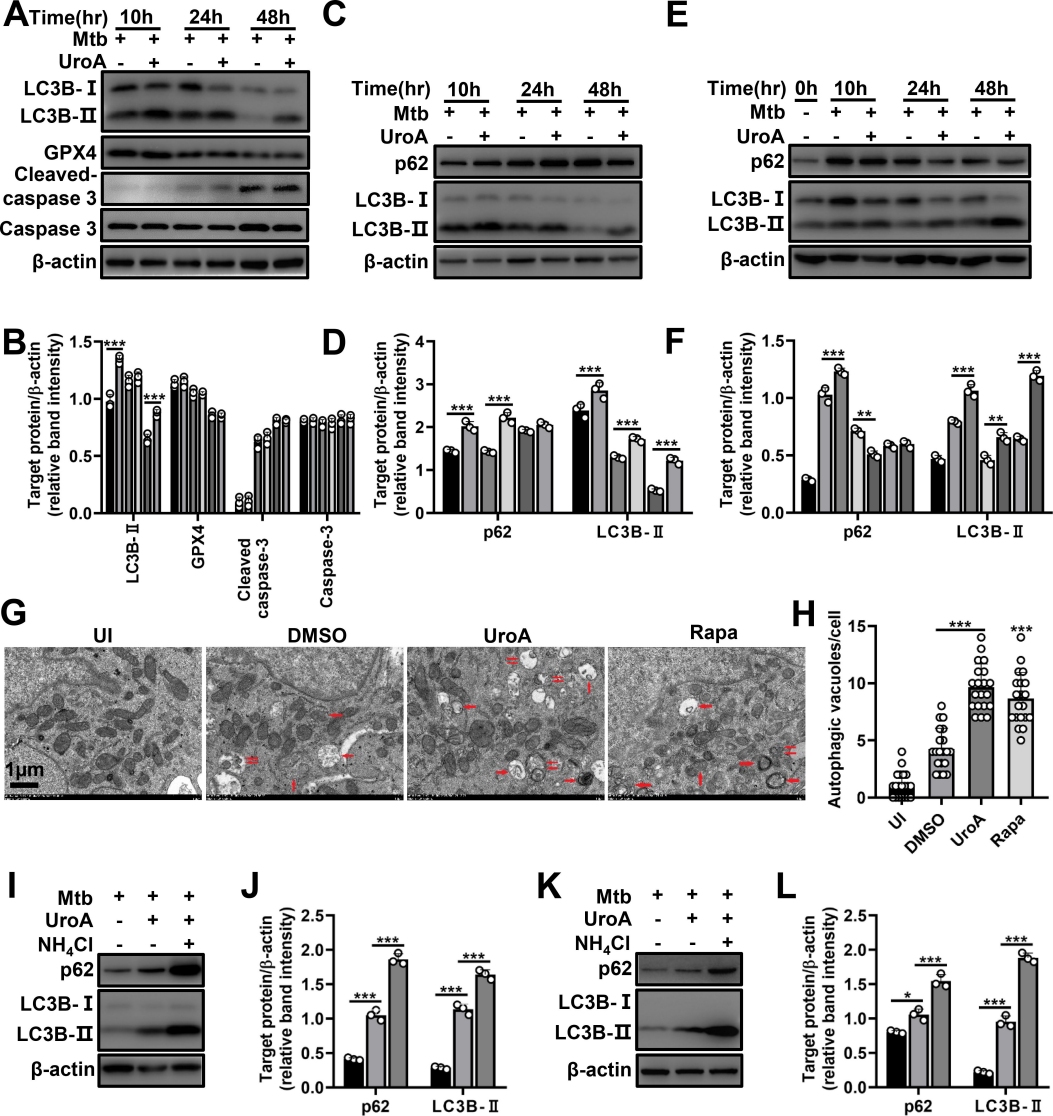
UroA enhances autophagy flux within Mtb-infected macrophages. (**A**) THP-1 cells were pretreated with UroA (60 µM) for 1 h and then infected with Mtb H37Rv (MOI, 10:1). Cell lysates were subjected to western blot analysis. β-Actin was used as a loading control. (**B**) The relative band intensities (target protein/β-actin) of proteins. (C–F) THP-1 cells (**C**) and BMDMs (**E**) were pretreated with UroA and infected with Mtb H37Rv as described in panel **A**. Cell lysates were subjected to western blot analysis using anti-p62, anti-LC3B, or anti-β-actin antibodies. (**D and F**) Relative band intensities. (**G**) THP-1 cells were pretreated with UroA (60 µM) for 1 h and then infected with Mtb H37Rv (MOI, 10:1) for 24 h. The cells were harvested, fixed, and then subjected to TEM analysis. One representative TEM image was shown (scale bar 1 µm). Single arrows indicate autolysosomes, and double arrows indicate autophagosomes. Rapamycin (Rapa) treatment was used as a positive control. (**H**) The number of autophagic vacuoles per cell was quantified within 20 cells in each sample. (I–L) THP-1 cells (**I**) and BMDMs (**K**) were pretreated with UroA and infected with Mtb H37Rv as described in panel **A** in the presence or absence of NH_4_Cl. Cell lysates were subjected to western blot analysis using anti-p62, anti-LC3B, or anti-β-actin antibodies. (**J and L**) Relative band intensities. For each target protein, the relative band intensities from left to right matched the different treatment conditions in the same order. The data are presented as mean ± SD of three independent experiments. Unless indicated otherwise, there is no significant difference; *P < 0.05; **P < 0.01; ***P < 0.001.

Autophagy plays an important part in the host fighting Mtb infection, and UroA can regulate autophagy to exert its beneficial effects (13). In the present study, we evaluated UroA-regulated autophagy within Mtb-infected macrophages. The transformation of lipid-bound light chain 3B (LC3B)-II from LC3B-I was significantly enhanced in UroA-treated THP-1 cells and BMDMs infected with Mtb (Fig. 2A through F; Fig. S1A through C). The level of p62, another autophagic marker, was significantly altered in both UroA-treated and Mtb-infected THP-1 cells and BMDMs (Fig. 2C through F; Fig. S1B and C). Besides, transmission electron microscope (TEM) results displayed elevated autophagic vacuoles in UroA-treated and Mtb-infected THP-1 cells compared to either uninfected ordimethyl sulfoxide (DMSO)-treated and Mtb-infected groups (Fig. 2G and H). These results suggest that UroA regulates autophagy during Mtb infection. In addition, no significant differences in the levels of glutathione peroxidase 4, cleaved caspase-3, or caspase-3 indicated that UroA did not affect ferroptosis or apoptosis (Fig. 2A and B; Fig. S1A). We further demonstrated whether UroA induced autophagic flux in Mtb-infected macrophages. NH_4_Cl is an autophagic inhibitor that reduces p62 and LC3B-II degradation through suppressing lysosomal acidification, and the levels of p62 and LC3B-II accumulation positively correlate with autophagic flux induction. As expected, NH_4_Cl preincubation further increased accumulation of LC3B-II and p62 in UroA-treated and Mtb-infected THP-1 cells and BMDMs compared with that of UroA-treated alone (Fig. 2I through L; Fig. S1D and E). These results suggest that UroA activates autophagic flux.

### UroA reduces Mtb survival by promoting autophagy *in vitro* and *in vivo*

**Fig 3:**
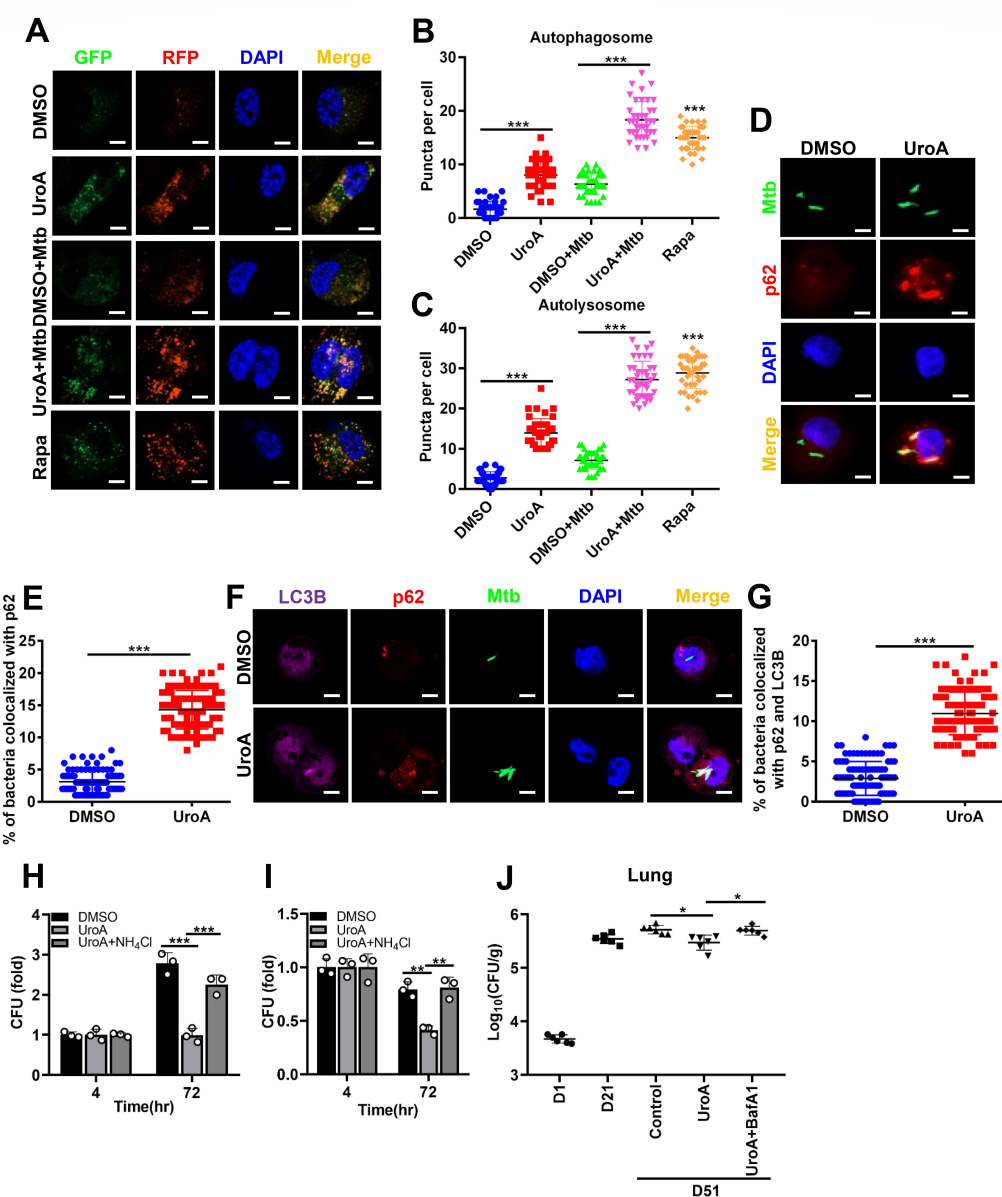
UroA reduces Mtb survival by promoting autophagy *in vitro* and *in vivo*. (A–C) THP-1 cells expressing mRFP-GFP-LC3B reporter were pretreated with UroA (60 µM) and infected with Mtb H37Rv (MOI, 10:1). Representative confocal microscopic image in panel **A**; bar, 5 µm. Rapamycin (Rapa) was used as a positive control. (**B and C**) Autophagosomal (yellow, **B**) and autolysosomal (red, **C**) puncta in panel **A** were counted. (**D and E**) THP-1 cells were pretreated and infected as described in Fig. 2A using a GFP-overexpressing Mtb. Cells were fixed and immunostained for p62. Representative confocal microscopic image in panel **D**; (bar, 5 µm). (**E**) Percent colocalization of Mtb with p62 within THP-1 cells. A total of 100 bacterial cells were counted. (**F and G**) BMDMs were pretreated and infected as described in Fig. 2A using a GFP-overexpressing Mtb. Cells were fixed and immunostained for p62 and LC3B. Representative confocal microscopic image in panel **F**; (bar, 5 µm). (**G**) Percent colocalization of Mtb with p62 and LC3B within BMDMs. A total of 100 bacterial cells were counted. (**H and I**) THP-1 cells (**H**) or BMDMs (**I**) were pretreated with UroA and infected with Mtb H37Rv, as shown in Fig. 2A in the presence or absence of NH_4_Cl. After washing thrice with warm phosphate-buffered saline, cells were treated with UroA for another 72 h in the presence or absence of NH_4_Cl. The number of CFUs was counted. As the difference is sufficient at 72 h postinfection, we did not perform the experiment at 24 and 48 h postinfection. (**J**) C57BL/6 mice were infected with ~200 CFU of H37Rv through aerosol. The infected mice were intragastric administration of either 0.5% sodium carboxymethyl cellulose (Control) or 0.5% sodium carboxymethyl cellulose harboring UroA/UroA + BafA1 at 3 weeks postinfection for one month (once every two days). The bacteria burden in the lungs was determined at 1 day (**D**), 21 days, and 51 days. The data are presented as mean ± SD of three independent experiments. Unless indicated otherwise, there is no significant difference; *P < 0.05; **P < 0.01; ***P < 0.001.

We further investigated UroA-induced autophagic flux by assessing LC3 enrichment within autophagosomes and autolysosomes using mRFP-GFP-LC3B reporter THP-1 cells. UroA treatment augmented autophagosomal and autolysosomal punctate formation in uninfected and Mtb-infected THP-1 cells compared with that in their respective controls, suggestive of autophagic flux induction (Fig. 3A through C). In addition, UroA increased xenophagy, as demonstrated by the improved co-localization of Mtb and p62 in THP-1 cells (Fig. 3D and E). Similarly, we observed increased co-localization of Mtb with p62 and LC3B in BMDMs (Fig. 3F and G). Next, the relationship of UroA-mediated autophagic flux and mycobactericidal effect was investigated. The UroA-mediated antimycobacterial effect was significantly impaired in Mtb-infected macrophages pretreatment with NH_4_Cl (Fig. 3H and I). In an Mtb-infected mouse model, UroA administration decreased bacterial burden in the lungs at day 51 in comparison to that of the control group; however, this effect was significantly counteracted in the presence of bafilomycin A1 (BafA1), an autophagic inhibitor that prevents the fusion of autophagosome and lysosome and lysosomal acidification (Fig. 3J). These data imply that UroA-induced elevation of autophagic flux contributes to the elimination of intracellular Mtb *in vitro* and *in vivo*.

### UroA-mediated autophagy is closely associated with protein kinase B (AKT) activity

**Fig 4:**
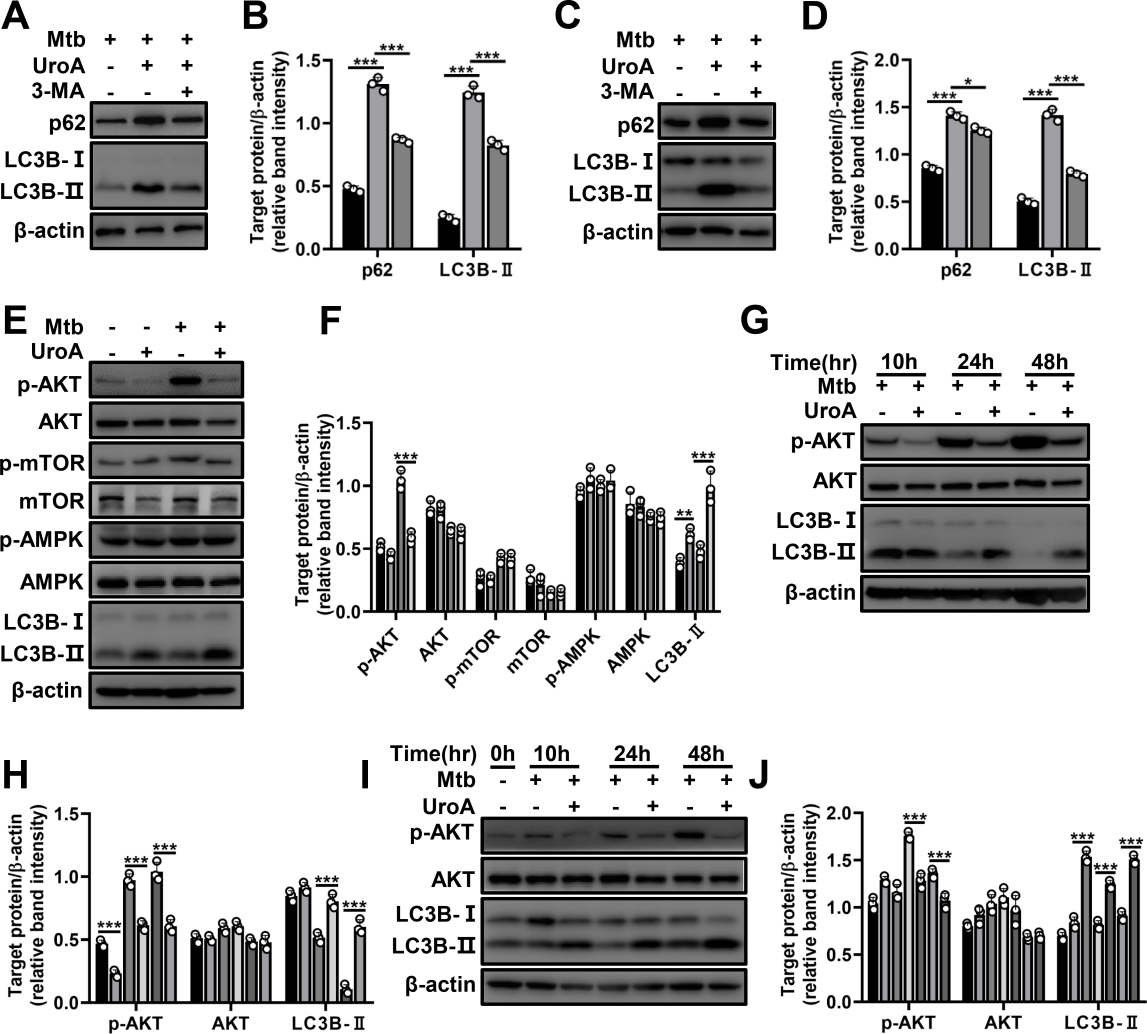
UroA-mediated autophagy is closely associated with AKT activity. (A–D) THP-1 cells (**A**) and BMDMs (**C**) were pretreated with UroA and infected with Mtb H37Rv as described in Fig. 2A in the presence or absence of 3-MA. Cell lysates were subjected to western blot analysis using anti-p62, anti-LC3B, or anti-β-actin antibodies. (**B and D**) Relative band intensities. (**E**) THP-1 cells incubated with UroA (60 µM) were infected with Mtb H37Rv (MOI, 10:1). Cell lysates were subjected to western blot analysis. (**F**) Relative band intensities. (G–J) THP-1 cells (**G**) and BMDMs (**I**) were pretreated with UroA and infected with Mtb H37Rv as described in Fig. 2A. Cell lysates were subjected to western blot analysis using anti-p-AKT, anti-AKT, anti-LC3B, or anti-β-actin antibodies. (**H and J**) Relative band intensities. For each target protein, the relative band intensities from left to right matched the different treatment conditions in the same order. The data are presented as mean ± SD of three independent experiments. Unless indicated otherwise, there is no significant difference; *P < 0.05; **P < 0.01; ***P < 0.001.

In order to clarify the underlying mechanism of UroA-triggered autophagy, we treated THP-1 cells and BMDMs with 3-methyladenine (3-MA), an inhibitor of autophagosome formation, and found that UroA-induced augmentation of LC3B-II and p62 was significantly reduced by 3-MA treatment, suggesting that UroA activated autophagy upstream of autophagosome formation (Fig. 4A through D; Fig. S2A and B). AKT, an upstream molecule of autophagosome formation, plays a crucial regulatory role in autophagy activation (21), and we hypothesized that AKT participated in UroA-triggered autophagy. The level of phosphorylated AKT (p-AKT) was suppressed in UroA-pretreated and uninfected or Mtb-infected THP-1 cells, whereas LC3B-II levels increased (Fig. 4E and F; Fig. S2C). The protein level of AKT was not obviously changed. AKT can regulate autophagy by affecting the phosphorylation of mammalian target of rapamycin (p-mTOR) and AMP-activated protein kinase (p-AMPK) (21). However, no significant differences were observed in the changes of p-mTOR, p-AMPK, and their protein levels (Fig. 4E and F; Fig. S2C). Besides, decreased p-AKT and increased LC3B-II levels were observed at different time points in UroA-treated and Mtb-infected THP-1 cells and BMDMs (Fig. 4G through J; Fig. S2D and E). The protein level of AKT was not significantly changed (Fig. 4G through J; Fig. S2D and E). These results imply that UroA activates autophagy by inhibiting AKT.

### AKT agonist inhibits UroA-mediated autophagy and antimycobacterial effect

**Fig 5:**
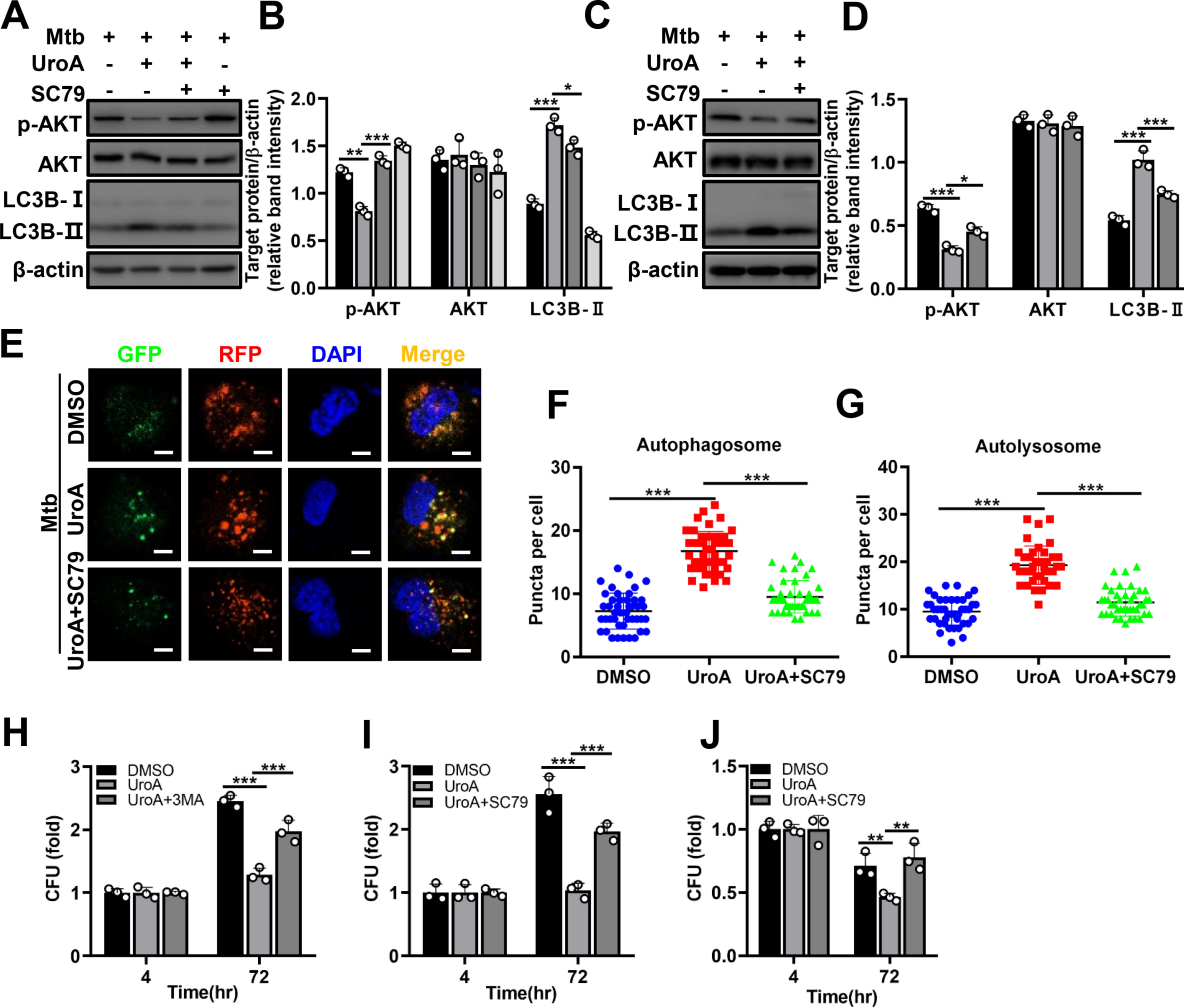
AKT agonist inhibits UroA-mediated autophagy and antimycobacterial effect. (A–D) THP-1 cells (**A**) and BMDMs (**C**) were pretreated with UroA and infected with Mtb H37Rv as described in Fig. 2A in the presence or absence of SC79. Cell lysates were subjected to western blot analysis using anti-p-AKT, anti-AKT, anti-LC3B, or anti-β-actin antibodies. (**B and D**) Relative band intensities. (E–G) THP-1 cells expressing mRFP-GFP-LC3B reporter were pretreated with UroA (60 µM) and infected with Mtb H37Rv (MOI, 10:1) in the presence or absence of SC79. Representative confocal microscopic image in panel **E**; (bar, 5 µm). (**F and G**) Autophagosomal (yellow, **F**) and autolysosomal (red, **G**) puncta in panel **E**. (H–J) THP-1 cells (**H and I**) or BMDMs (**J**) were pretreated with UroA and infected with Mtb H37Rv as described in Fig. 2A in the presence or absence of 3-MA or SC79. After washing with warm phosphate-buffered saline, cells were retreated with UroA for another 72 h in the presence or absence of 3-MA or SC79. The number of CFUs was counted. As the difference is sufficient at 72 h postinfection, we did not perform the experiment at 24 and 48 h postinfection. For each target protein, the relative band intensities from left to right matched the different treatment conditions in the same order. The data are presented as mean ± SD of three independent experiments. Unless indicated otherwise, there is no significant difference; *P < 0.05; **P < 0.01; ***P < 0.001.

We further investigated the role of AKT in UroA-mediated autophagy using SC79, an AKT agonist. UroA-mediated inhibition of AKT phosphorylation and LC3B-II augmentation were reversed in Mtb-infected THP-1 cells and BMDMs in the presence of SC79 (Fig. 5A through D; Fig. S3A and B). The protein level of AKT was not significantly changed (Fig. 5A through D; Fig. S3A and B). Similarly, SC79 pretreatment significantly reduced UroA-induced autophagosomal and autolysosomal puncta (Fig. 5E through G). The results suggest that SC79 preincubation inhibits UroA-induced autophagy. In addition, preincubation with 3-MA and SC79 significantly decreased the antimycobacterial effects of UroA (Fig. 5H through J). Therefore, UroA-induced autophagy exerts antimycobacterial effects by inhibiting AKT activation and its activity.

### UroA activates autophagy by regulating AKT–forkhead box protein O1 (FOXO1) signaling

**Fig 6:**
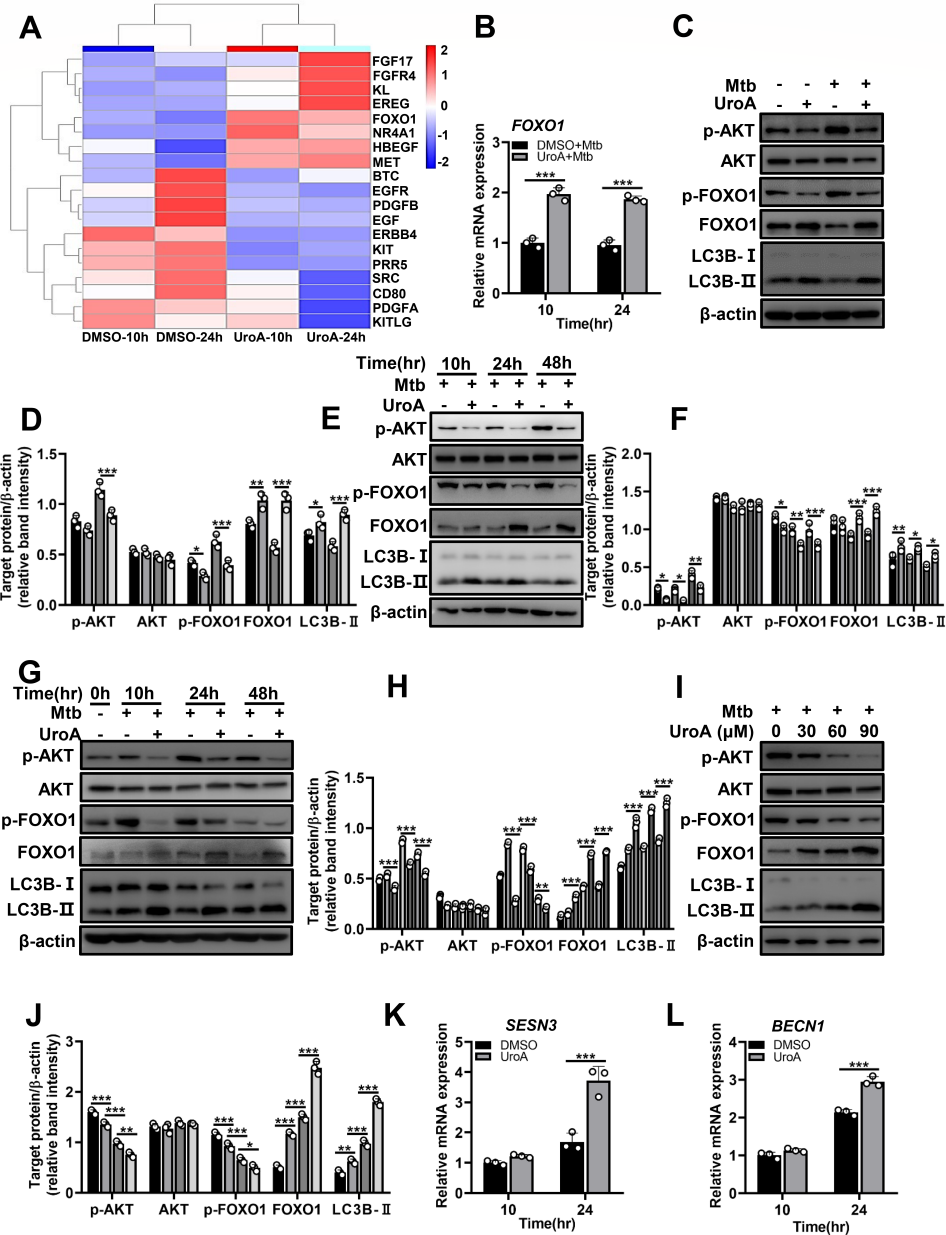
UroA activates autophagy by regulating AKT–FOXO1 signaling. (**A**) Genes involved in the PI3K–AKT signaling pathway in UroA-treated and Mtb H37Rv-infected THP-1 cells assessed by the Kyoto Encyclopedia of Genes and Genomes pathway enrichment analysis. (**B**) The relative expression of *FOXO1* in Mtb H37Rv-infected THP-1 cells with or without UroA treatment. (**C**) THP-1 cells treated with UroA (60 µM) were infected with Mtb H37Rv (MOI, 10:1). Cell lysates were subjected to western blot analysis using anti-p-AKT, anti-AKT, anti-p-FOXO1, anti-FOXO1, anti-LC3B, or anti-β-actin antibodies. (**D**) Relative band intensities. (E–H) THP-1 cells (**E**) and BMDMs (**G**) were pretreated with UroA and infected with Mtb H37Rv as described in Fig. 2A. Cell lysates were subjected to western blot analysis. (**F and H**) Relative band intensities. (**I**) THP-1 cells were preincubated with UroA at different concentrations (0 µM–90 µM) and then infected with Mtb H37Rv (MOI, 10:1). Cell lysates were subjected to western blot analysis. (**J**) Relative band intensities. (**K and L**) Relative expression of *SESN3* (**K**) and *BECN1* (**L**) in Mtb H37Rv-infected THP-1 cells with or without UroA treatment. For each target protein, the relative band intensities from left to right matched the different treatment conditions in the same order. The data are presented as mean ± SD of three independent experiments. Unless indicated otherwise, there is no significant difference; *P < 0.05; **P < 0.01; ***P < 0.001.

To further explore the mechanisms of UroA-mediated modulation of autophagy, RNA sequencing (RNA-seq) analysis was performed in Mtb-infected THP-1 cells with or without pretreatment of UroA. After pathway enrichment analysis, genes related to the phosphatidylinositol-3-kinase (PI3K)–AKT signaling were discovered to be significantly differentially expressed between dimethyl sulfoxide- and UroA-treated groups (Fig. 6A). Among these, upregulated expression of FOXO1, which is associated with the modulation of autophagy, attracted our attention. Quantitative real-time polymerase chain reaction (qRT-PCR) and western blot analysis indicated increased expression of FOXO1 in UroA-pretreated uninfected or Mtb-infected macrophages compared with their respective controls (Fig. 6B through D; Fig. S4A). AKT promotes FOXO1 phosphorylation and inhibits FOXO1 nucleus translocation and transcriptional activation (28). Interestingly, UroA treatment significantly decreased p-AKT and p-FOXO1 levels and increased LC3B-II levels in uninfected and Mtb-infected macrophages compared with those in their respective controls (Fig. 6C and D; Fig. S4A). The protein level of AKT was not obviously changed (Fig. 6C and D; Fig. S4A). Moreover, this trend was observed at different time points in UroA-treated THP-1 cells and BMDMs infected with Mtb (Fig. 6E through H; Fig. S4B and C). The protein level of AKT was not obviously changed (Fig. 6E through H; Fig. S4B and C). Besides, UroA dose-dependently reduced p-AKT and p-FOXO1 levels and elevated FOXO1 and LC3B-II levels during Mtb infection (Fig. 6I and J; Fig. S4D). The protein level of AKT was not obviously changed (Fig. 6I and J; Fig. S4D). Given that FOXO1 is an autophagy-regulating transcription factor, we further analyzed the RNA-seq data and found that the FOXO1-regulated transcription of *Sestrin-3* (*SESN3*) and *Beclin-1* (*BECN1*) was significantly upregulated in UroA-treated and Mtb-infected macrophages compared with that in their respective controls (Table 2). qRT-PCR validation showed similar results (Fig. 6K and L). These results indicate that UroA induces autophagy by targeting AKT–FOXO1 signaling.

**TABLE 2:** UroA stimulated the upregulation of autophagy-related genes regulated by FOXO1

Gene name	UroA/DMSO (10 h)		UroA/DMSO (24 h)	
	Log2 fold change	*P*-value	Log2 fold change	*P*-value
*SESN3*	0.81	0.0009	0.98	0.0004
*BECN1*	0.23	0.02	0.26	0.005

### AKT–FOXO1 signaling regulated by UroA activates autophagy to exert antimycobacterial effects

**Fig 7:**
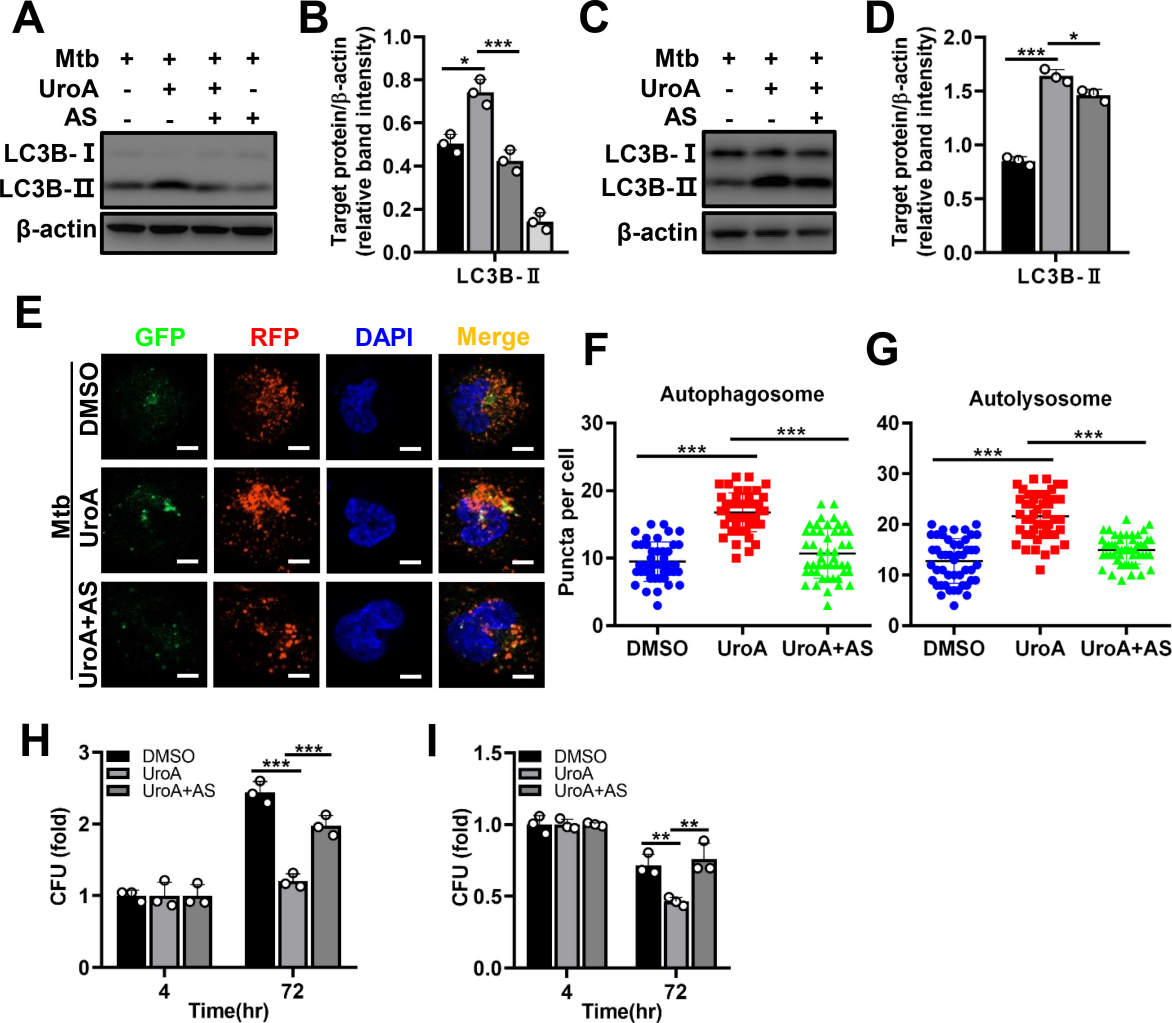
AKT–FOXO1 signaling regulated by UroA activates autophagy to exert antimycobacterial effects. (A–D) THP-1 cells (**A**) and BMDMs (**C**) were pretreated with UroA and infected with Mtb H37Rv as described in Fig. 2A in the presence or absence of AS. Cell lysates were subjected to western blot analysis. (**B and D**) Relative band intensities. (E–G) THP-1 cells expressing mRFP-GFP-LC3B reporter were pretreated with UroA (60 µM) and infected with Mtb H37Rv (MOI, 10:1) in the presence or absence of AS. Representative confocal microscopic image in panel **E**; (bar, 5 µm). (**F and G**) Autophagosomal (yellow, **F**) and autolysosomal (red, **G**) puncta in panel **E**. (**H and I**) THP-1 cells (**H**) or BMDMs (**I**) were pretreated with UroA and infected with Mtb H37Rv as described in Fig. 2A in the presence or absence of AS. After washing with warm phosphate-buffered saline, cells were retreated with UroA for another 72 h in the presence or absence of AS. The number of CFUs was counted. As the difference is sufficient at 72 h postinfection, we did not perform the experiment at 24 and 48 h postinfection. For each target protein, the relative band intensities from left to right matched the different treatment conditions in the same order. The data are presented as mean ± SD of three independent experiments. Unless indicated otherwise, there is no significant difference; *P < 0.05; **P < 0.01; ***P < 0.001.

The role of FOXO1 activity in UroA-induced autophagy was further demonstrated using AS1842856 (AS), a specific inhibitor of FOXO1. Pretreatment with AS significantly reduced the UroA-induced LC3B-II expression in Mtb-infected THP-1 cells and BMDMs (Fig. 7A through D; Fig. S5A and B). Moreover, AS decreased the number of autophagosomal and autolysosomal puncta induced by UroA during Mtb infection (Fig. 7E through G). Consistently, the antimycobacterial effect of UroA was significantly reduced in Mtb-infected THP-1 cells and BMDMs in the presence of AS (Fig. 7H and I). Collectively, our data suggest that UroA inhibits intracellular Mtb growth by regulating AKT–FOXO1-mediated autophagy.

### Cotreatment of INH with UroA synergistically contributes to Mtb clearance

**Fig 8:**
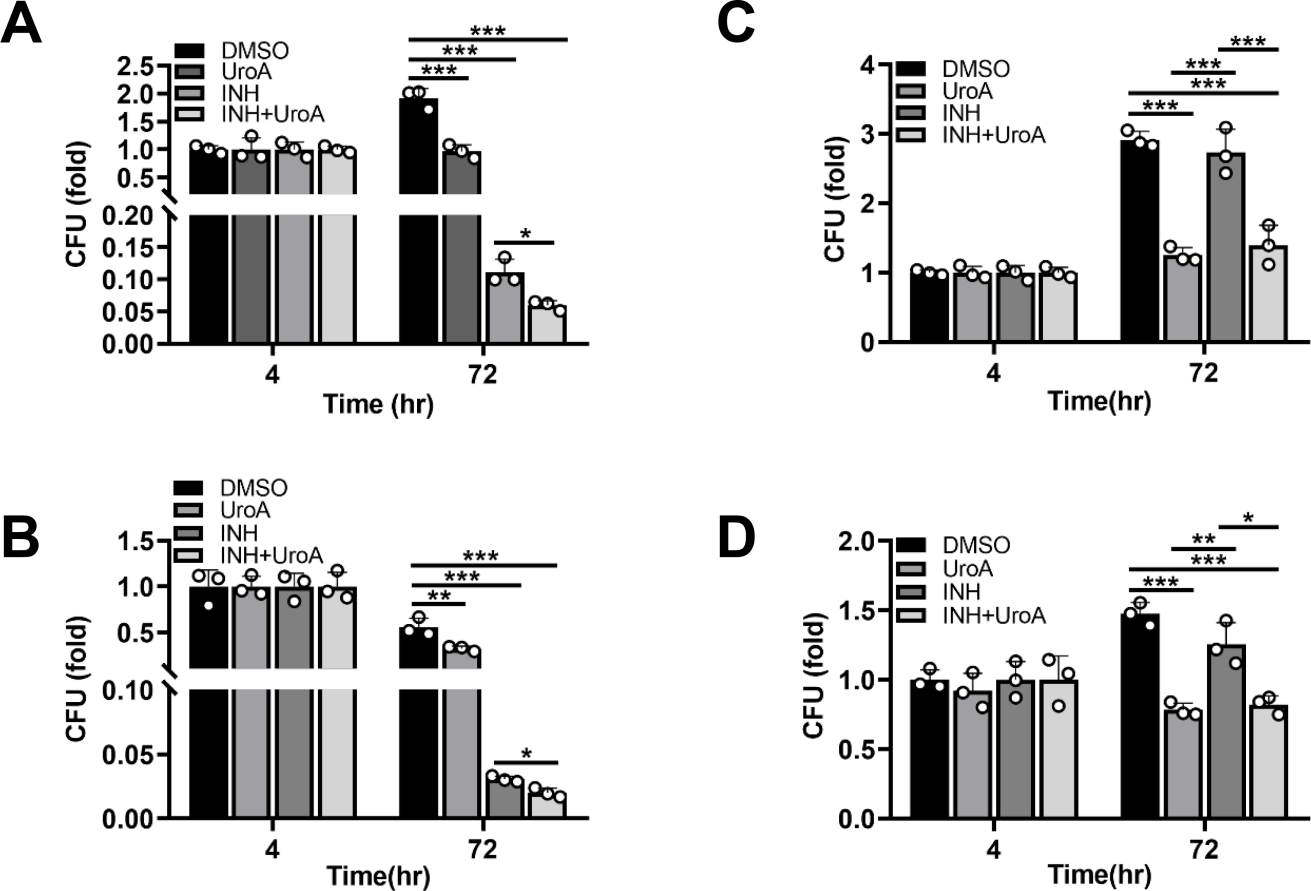
Combined utilization of INH and UroA synergistically contributes to Mtb clearance. (**A and B**) THP-1 cells (**A**) and BMDMs (**B**) preincubated with UroA for 1 h were infected with Mtb H37Rv (MOI, 10:1) for 4 h. After washing thrice with warm phosphate-buffered saline (PBS), the infected macrophages were incubated with UroA (60 µM) in combination with INH (0.1 µg/mL) for another 72 h. Cells were lysed, and the number of CFUs was counted. As the difference is sufficient at 72 h postinfection, we did not perform the experiment at 24 and 48 h postinfection. (**C and D**) THP-1 cells (**C**) and BMDMs (**D**) preincubated with UroA for 1 h were infected with C2 (MOI, 10:1) for 4 h. After washing thrice with warm PBS, the infected macrophages were incubated with UroA (60 µM) in combination with INH (0.1 µg/mL) for another 72 h. Cells were lysed, and the number of CFUs was counted. As the difference is sufficient at 72 h postinfection, we did not perform the experiment at 24 and 48 h postinfection. The data are presented as mean ± SD of three independent experiments. Unless indicated otherwise, there is no significant difference; *P < 0.05; **P < 0.01; ***P < 0.001.

The above results showed that UroA may be a potential HDT candidate, and the combined effect of UroA and INH (a first-line anti-TB drug) against Mtb infection was analyzed. Cotreatment of INH and UroA significantly restricted intracellular Mtb H37Rv in both THP-1 cells and BMDMs compared with that by single treatment of INH or UroA, as evident from the number of CFUs (Fig. 8A and B). Moreover, UroA combined with INH or UroA alone significantly suppressed the growth of INH-resistant clinical Mtb (C2) in THP-1 cells and BMDMs (Fig. 8C and D). Collectively, these results suggest that UroA may be used as a possible adjunctive treatment with first-line anti-TB drugs for treating TB, particularly drug-resistant TB.

## DISCUSSION

UroA, a metabolite transformed by gut microbes, exerts multiple beneficial effects (13). However, the antimycobacterial activity and underlying mechanisms of action of UroA remain unclear. Here, UroA effectively inhibited the intracellular growth of Mtb H37Rv and clinically INH-resistant Mtb by inducing autophagy via regulating AKT–FOXO1 signaling.

UroA inhibits the proliferation of enterovirus 71 and the growth of *Helicobacter pylori* (26, 27). Consistent with these observations, we found that Mtb survival within macrophages was significantly reduced by UroA treatment. UroA-induced autophagy is closely associated with multiple host protective functions such as anti-inflammatory, antitumor, and anti-aging effects (13). Similarly, we found that UroA-induced autophagy facilitated its antimycobacterial effects. Notably, AKT signaling plays a vital role in UroA-induced autophagy. UroA upregulates autophagy by impairing AKT–mTOR signaling, contributing to its anti-inflammatory and antidiabetic potential (21, 29). UroA promotes cytoprotective autophagy by downregulating PI3K–AKT–mTOR or AKT–serine/threonine-protein kinase WNK1 signaling, thereby exerting antitumor effects (15, 23). Consistently, we found that UroA triggered autophagy by reducing the phosphorylation and activity of AKT during Mtb infection. AKT is a critical downstream molecule of PI3K signaling and is crucial in determining cell fate (30). In classical PI3K–AKT signaling, phosphoinositide-dependent kinase-1 catalyzes AKT1 phosphorylation at Thr308 on the internal surface of the cell membrane (31). Active AKT translocates to other cellular subcomponents to trigger downstream signaling by regulating the activity of multiple substrates, including the transcription factor FOXO1 (32). AKT initiates FOXO1 phosphorylation, causing the nuclear export of FOXO1 and its subsequent degradation through the ubiquitin-mediated proteasomal pathway (28). UroA treatment significantly decreased FOXO1 phosphorylation in Mtb-infected macrophages. Dephosphorylated FOXO1 was translocated to the nucleus and exerted its transcriptional activity. FOXO1 is a key mediator of autophagy and is closely involved in the regulation of autophagy-associated genes, including *SESN3* and *BECN1* (33). FOXO1-induced SESN3 expression suppresses the mammalian target of rapamycin complex 1 activity through tuberin-dependent mechanisms, which promotes autophagy (34, 35). FOXO1-induced BECN1 expression facilitates autophagosome formation via the BECN1–PI3K complex (36). Similarly, our results showed that UroA stimulation significantly enhanced the transcription of *SESN3* and *BECN1* in Mtb-infected macrophages. FOXO1 triggers autophagy by transactivating multiple autophagy-related genes (37). However, in the present study, UroA treatment did not significantly upregulate the expression of autophagy-related genes during Mtb infection, which might be condition-specific. Moreover, the AKT agonist and FOXO1 inhibitor significantly counteracted UroA-mediated antimycobacterial effects. Collectively, our results suggest that UroA induces autophagy by regulating AKT–FOXO1 signaling-mediated expression of pro-autophagy genes, which contributes to the antimycobacterial activity of UroA.

Importantly, oral administration of synthetic UroA is non-genotoxic to rats (38). Moreover, no undesired side effects have been observed in 28- and 90-day oral studies (38). Oral consumption of UroA has a beneficial safety profile in healthy and sedentary older individuals (39). Moreover, plasma acylcarnitine and skeletal muscle mitochondrial gene expression have been found to be regulated by UroA treatment (39). In a randomized, placebo-controlled trial involving middle-aged adults, oral administration of UroA has been found to be safe and significantly enhanced muscle strength, aerobic endurance, and physical performance (40). These results suggest that UroA can be safely consumed for clinical and therapeutic purposes.

HDT, as an adjunctive treatment in combination with anti-TB drugs, is an effective means of treating drug-resistant TB. The addition of CC214-2, an inhibitor of mTOR kinase, to the first-line or bedaquiline–pretomanid–linezolid regimen decreases relapses in Mtb-infected mice (41). The combined use of imatinib and first-line drugs exhibits synergistic and antimycobacterial activity, and imatinib alone contributes to the elimination of rifampicin-resistant strains (42). Interestingly, berberine, a natural compound derived from medicinal plants, promotes host clearance of drug-sensitive and drug-resistant TB by regulating the functions of macrophages and Th1/Th17 and T cells (43). Moreover, berberine significantly reduces the risk of TB relapse by targeting neurogenic locus notch homolog protein 3–phosphatidylinositol 3,4,5-trisphosphate 3-phosphatase and dual-specificity protein phosphatase PTEN–AKT–FOXO1 signaling (44). In addition, berberine co-administered with rifampin and INH reduces Mtb burden within the lungs and spleen and relieves histological damage (45). Other natural products, including piperine, curcumin, bergenin, and luteolin, also show excellent potential for HDT, either alone or in combination with antimycobacterial drugs against drug-sensitive and drug-resistant Mtb (46). Similarly, our results demonstrated that the combined use of UroA and INH resulted in better antibacterial effects than that of INH alone. Moreover, UroA significantly inhibited the intracellular survival of a clinically INH-resistant strain. Therefore, plant-based natural compounds, such as UroA, may be utilized as HDT drugs for adjuvant therapy in TB.

In summary, it may be utilized as a potential HDT candidate and utilized as an adjunctive treatment with first-line antimycobacterial drugs.

## MATERIALS AND METHODS

### Materials

UroA, SC79, AS, BafA1, and rapamycin were obtained from MedChemExpress (Monmouth Junction, USA). INH and NH_4_Cl were obtained from Sangon (Shanghai, China) and Sigma (USA), respectively, and dissolved in deionized water. The solution was sterile-filtered using a 0.22 µm filter (Millipore, Bedford, USA).

### C57BL/6 mice

BMDMs were obtained from 8-week-old female mice.

### Cell culture

Human acute monocytic leukemia cell line (THP-1) was obtained from the Cell Bank of the Chinese Academy of Sciences (Shanghai, China), and THP-1 cells stably expressing an mRFP-GFP-LC3B reporter (kindly provided by Professor Xinchun Chen, Shenzhen University, Shenzhen, China) were grown in Roswell Park Memorial Institute (RPMI) 1640 (Gibco, NY, USA) containing 10% fetal bovine serum (Invitrogen, Life Technologies, Grand Island, NY), 1% 4-(2-hydroxyethyl)-1-piperazineethanesulfonic acid (Gibco), and 1% sodium pyruvate (Gibco) (complete medium) at 37°C in an incubator with 5% CO_2_. Phorbol-12-myristate-13-acetate (50 ng/mL; Sigma-Aldrich) was used to stimulate the differentiation of THP-1 cells into macrophages. After stimulation for 48 h, the culture medium was removed and replaced with fresh and complete RPMI 1640 medium for another 24 h.

### Mycobacterium strains and culture conditions

Mtb H37Rv and clinically INH-resistant Mtb (C2) were cultivated in Middlebrook 7H9 broth (Difco, BD Biosciences, San Jose, CA, USA) supplemented with 10% oleic acid-albumin-dextrose-catalase (OADC; Becton Dickinson, MD, USA) and 0.05% Tween 80 at 37°C. Bacterial cells from the mid-log phase were centrifuged, washed, and resuspended in serum-free RPMI 1640, which was used to infect macrophages.

### Cell counting kit-8 (CCK8) assay

THP-1 cells (2 × 10^4^ cells/well) were preincubated with UroA at different concentrations (0, 10, 30, 60, 100, 120, 180, 210, 240, and 270 µM) for 1 h and then infected with Mtb H37Rv at an MOI of 10 (10:1) for 4 h. After washing thrice with warm phosphate-buffered saline (PBS), cells were re-incubated with UroA for another 72 h. Then, cells were incubated with CCK8 solution for 2 h. Absorbance at 450 nm was determined using a spectrophotometer (Varioskan LUX, Thermo Scientific, USA).

### CFU counting

THP-1 cells (2.5 × 10^5^ cells) were preincubated with UroA (60 µM) for 1 h and then infected with H37Rv or C2 (MOI, 10:1) for 4 h at 37°C in an incubator with 5% CO_2_. Cells were washed thrice with warm PBS to remove extracellular bacteria and then retreated with UroA for another 72 h. Cells were lysed, diluted, and spread on Middlebrook 7H10 agar plates containing 10% OADC. The number of CFUs was counted after 3 weeks of growth at 37°C.

### Confocal microscopy

mRFP-GFP-LC3B-expressing THP-1 cells, THP-1 cells, or BMDMs preincubated with UroA (60 µM) for 1 h were infected with Mtb H37Rv or green fluorescent protein (GFP)-overexpressing Mtb H37Rv. After washing with PBS, macrophages were retreated with UroA for 24 h. After fixation with 4% paraformaldehyde for 10 min, infected mRFP-GFP-LC3B-expressing THP-1 cells were stained with 4′,6-diamidino-2-phenylindole (Beyotime, Nanjing, China) for 10 min at 24°C. THP-1 cells were incubated with an anti-p62 and/or anti-LC3B primary antibody and corresponding Alexa Fluor 594- and/or 633-conjugated secondary antibody for 2 and 1 h at 24°C, respectively. After washing with PBS, cells were imaged using an LSM700 microscope (Carl Zeiss, Oberkochen, Germany). The images were analyzed using ZEN software. A total of 40–50 infected cells were analyzed, and changes in autophagy were examined by enumerating LC3 forming puncta per cell. In addition, the percentage of co-localization of Mtb with p62 in THP-1 cells or Mtb with p62 and LC3B was determined. A total of 100 bacterial cells were counted.

### Transmission electron microscopy

The cells were harvested and treated according to the previous description (47). The images were taken using the Hitachi TEM system (Hitachi 7700, Japan).

### RNA-seq analysis

THP-1 cells pretreated with UroA and infected with Mtb H37Rv were lysed using TRIzol (Thermo Fisher Scientific, NY, USA). The samples were sent to Novogene (Guangzhou, China) for RNA-seq analysis.

### qRT-PCR

Total RNA isolated from UroA-treated and Mtb H37Rv-infected THP-1 cells was reverse-transcribed into cDNA using a HiScript IV RT SuperMix Kit (Vazyme, Nanjing, China). cDNA was used for qRT-PCR analysis on an Applied Biosystems 7500 system (Applied Biosystems, Foster City, CA, USA) with a SYBR Green PCR Kit (Vazyme). The primer pairs used were as follows: *FOXO1*, forward 5′-GATGGTCAAGAGCGTGCCCTAC-3′, and reverse, 5′-TGGATTGAGCATCCACCAAGAA-3′; *SESN3*, forward 5′-GAGGATGTTGACACAACCATGCTG-3′, and reverse, 5′-CCGCCAGTAACTATCATACATGCG-3′; *BECN1*, forward 5′-AGCTGCCGTTATACTGTTCTG-3′, and reverse, 5′- ACTGCCTCCTGTGTCTTCAATCTT-3′; and *GAPDH*, forward, 5′-ACCACAGTCC ATGCCATCAC-3′, and reverse, 5′-TCCACCACCC TGTTGCTGTA-3′. *GAPDH* was used as a housekeeping gene. Relative gene expression was normalized with respect to that of *GAPDH*. Fold changes were determined using the 2^-ΔΔCt^ method (48).

### Western blot analysis

THP-1 cells pretreated with UroA and infected with Mtb H37Rv were lysed using radio-immunoprecipitation assay (RIPA) lysis buffer (Beyotime). Equal volumes of cell lysates were subjected to sodium dodecyl sulfate-polyacrylamide gel electrophoresis and transferred to polyvinylidene fluoride (PVDF) membranes. Membranes were blocked using 5% nonfat milk for 1 h at 24°C and then incubated with antibodies against LC3B (Sigma-Aldrich, 1:1,000 dilution), β-actin (Cell Signaling Technology [CST], Berkeley, CA, USA, 1:10,000 dilution), p62 (Abcam, Cambridge, MA, USA, 1:1,000 dilution), p-FOXO1 (CST, 1:1,000 dilution), FOXO1 (CST, 1:1,000 dilution), p-AMPKα (CST, 1:1,000 dilution), AMPKα (CST, 1:1,000 dilution), GPX4 (CST, 1:1,000 dilution), cleaved caspase-3 (CST, 1:1,000 dilution), caspase 3 (CST, 1:1,000 dilution), p-AKT (CST, 1:1,000 dilution), AKT (CST, 1:1,000 dilution), p-mTOR (Abcam, 1:1,000 dilution), and mTOR (Abcam, 1:1,000 dilution) overnight at 4°C. After washing thrice with Tris-buffered saline containing 0.1% Tween 20, membranes were incubated with the corresponding secondary antibodies (CST, 1:10,000 dilution). Finally, protein bands were captured using a ChemiDoc MP imager (Bio-Rad, CA, USA).

### Isolation of BMDMs

Cells isolated from mouse bone marrow were stimulated in complete RPMI 1640 medium containing 20 µg/mL macrophage colony stimulating factor (PeproTech, Hartford, CT, USA) at 37°C in an incubator with 5% CO_2_. After 3 days, each dish was supplemented with an isometric complete RPMI 1640 medium containing 20 µg/mL macrophage colony-stimulating factor (M-CSF) for another 2 days. The medium was then removed. After washing with PBS, the adherent cells were restimulated with complete RPMI 1640 medium containing 20 µg/mL M-CSF for another 2 days. Well-differentiated macrophages were washed with PBS and treated with 0.25% trypsin. The cell suspension was used in further experiments.

### Mice infection and administration

C57BL/6 female mice (6–8 weeks old) were carried on aerosol infection of H37Rv (~200 CFU per mouse). The infected mouse was delivered drugs by gavage according to the doses below: 0.5% sodium carboxymethyl cellulose (MedChemExpress) only; 0.5% sodium carboxymethyl cellulose harboring UroA (50 mg/kg) or UroA + BafA1 (50 mg/kg + 1 mg/kg) at 3 weeks postinfection. Mice were killed at 1 day, 21 days, and 51 days post-infection. After lung homogenization, the homogenate was 10-fold dilution and plated on 7H10 medium. CFU were calculated after 4 weeks growth at a 37°C incubator.

### Statistical analysis

The data were analyzed using unpaired *t*-test, multiple *t*-test, one-way analysis of variance followed by Tukey’s test, or two-way analysis of variance followed by Bonferroni’s test. Data are presented as mean ± standard deviation and were processed using GraphPad Prism v.7 (GraphPad Software, Inc., La Jolla, CA, USA). A *P*-value <0.05 was considered significant.

## Data Availability

The raw data of RNA-seq has been submitted to the Sequence Read Archive of the National Center for Biotechnology Information (PRJNA1213988).

## SUPPLEMENTAL MATERIAL

Supplemental material msphere.00061-25-s0001.docx Figures S1 to S5.

ASM does not own the copyrights to Supplemental Material that may be linked to, or accessed through, an article. The authors have granted ASM a non-exclusive, world-wide license to publish the Supplemental Material files. Please contact the corresponding author directly for reuse.
