# Supplementary material for: Effect of urolithin A on intracellular survival of Mycobacterium tuberculosis by regulating AKT-FOXO1-mediated autophagy
Source: mSphere. 2025 Apr 10;10(5):e00061-25. doi: 10.1128/msphere.00061-25 (PMC12108056; doi:10.1128/msphere.00061-25)
Supplement: Supplemental material — Figures S1 to S5. [file msphere.00061-25-s0001.docx]

# cEffect of urolithin A on intracellular survival of *Mycobacterium tuberculosis* by regulating AKT-FOXO1-mediated autophagy

**Running title:** Gut microbe-derived urolithin A enhances TB treatment

Jing Bi ^1#^, Li Song ^1#^, Qinglong Guo ^1#^, Xi Chen ^2#^, Yaqi Gong ^3^, Haojia Wu ^1^, Fan Zhang ^1^, Jingbin Wang ^4*^, Guoliang Zhang ^1, 3*^

^1^ National Clinical Research Center for Infectious Diseases, Shenzhen Third People's Hospital, Southwest Medical University, Shenzhen, 518112, China

^2^ School of Public Health, Guangdong Medical University, Dongguan, 523808, China

^3^ School of Medicine, Southern University of Science and Technology, Shenzhen, 518055, China

^4^ Shenzhen Hospital of Guangzhou University of Chinese Medicine (Futian), Shenzhen, 518000, China

^#^ These authors have contributed equally to this work.

*Correspondence author

Dr. Guoliang Zhang, zhanggl2020@mail.sustech.edu.cn

Dr. Jingbin Wang, [kswon@qq.com](mailto:kswon@qq.com)


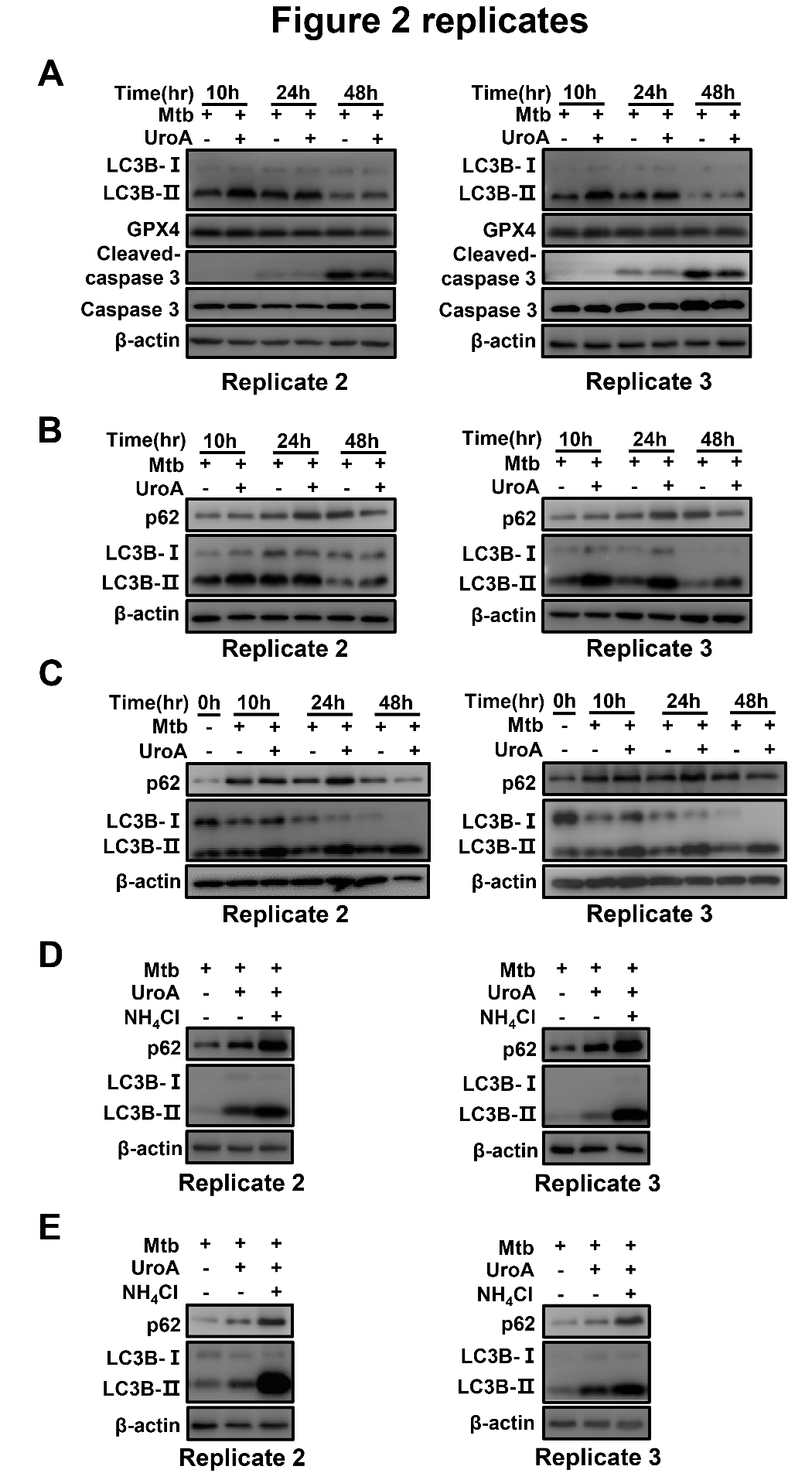
**Supporting Information**

**Supplementary Figure 1. Western blot replicates for Figure 2.** **(A)** Western blot replicates for Figure 2A. **(B)** Western blot replicates for Figure 2C. **(C)** Western blot replicates for Figure 2E. **(D)** Western blot replicates for Figure 2G. **(E)** Western blot replicates for Figure 2I.


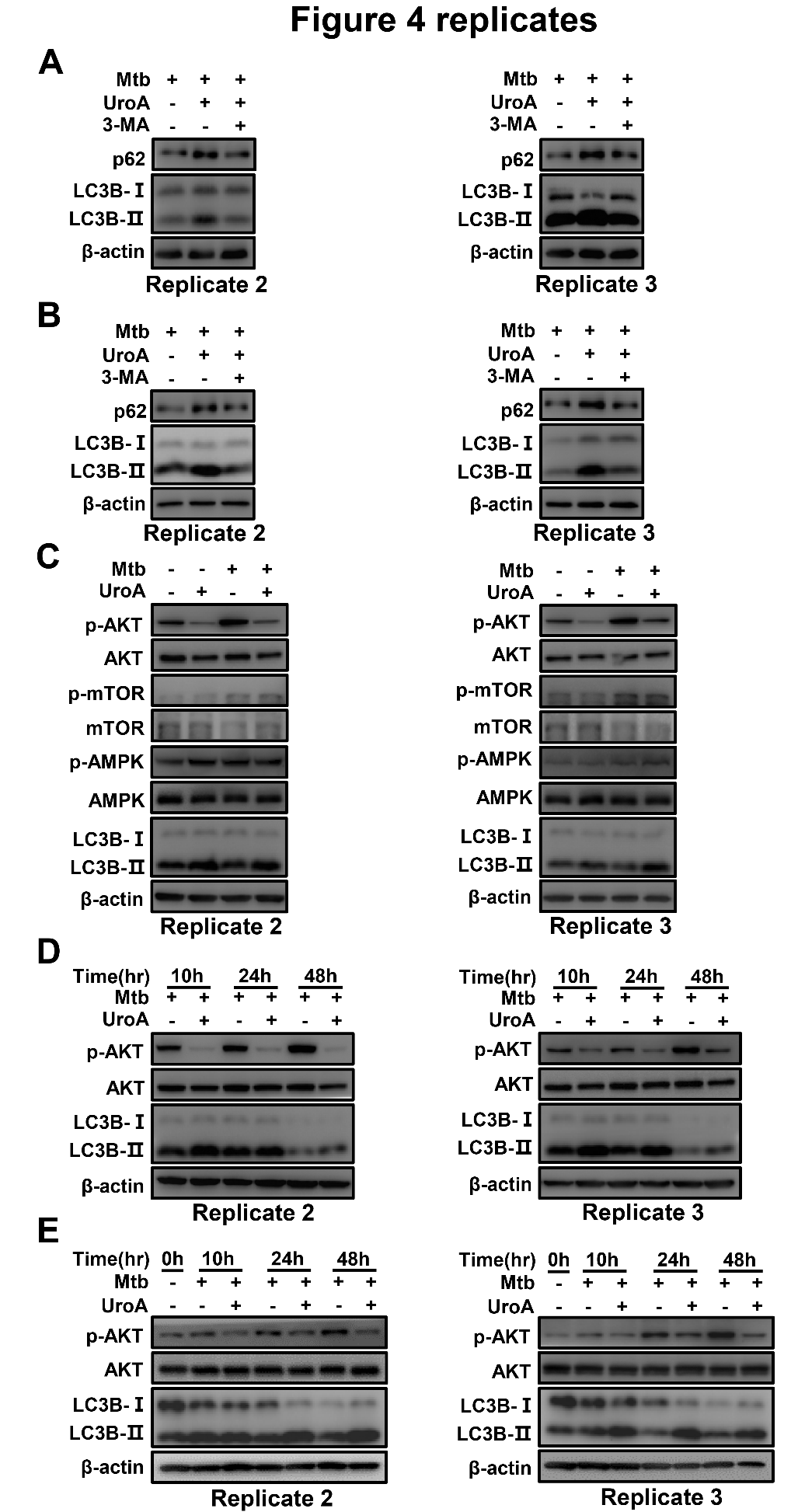


**Supplementary Figure 2. Western blot replicates for Figure 4.** **(A)** Western blot replicates for Figure 4A. **(B)** Western blot replicates for Figure 4C. **(C)** Western blot replicates for Figure 4E. **(D)** Western blot replicates for Figure 4G. **(E)** Western blot replicates for Figure 4I.


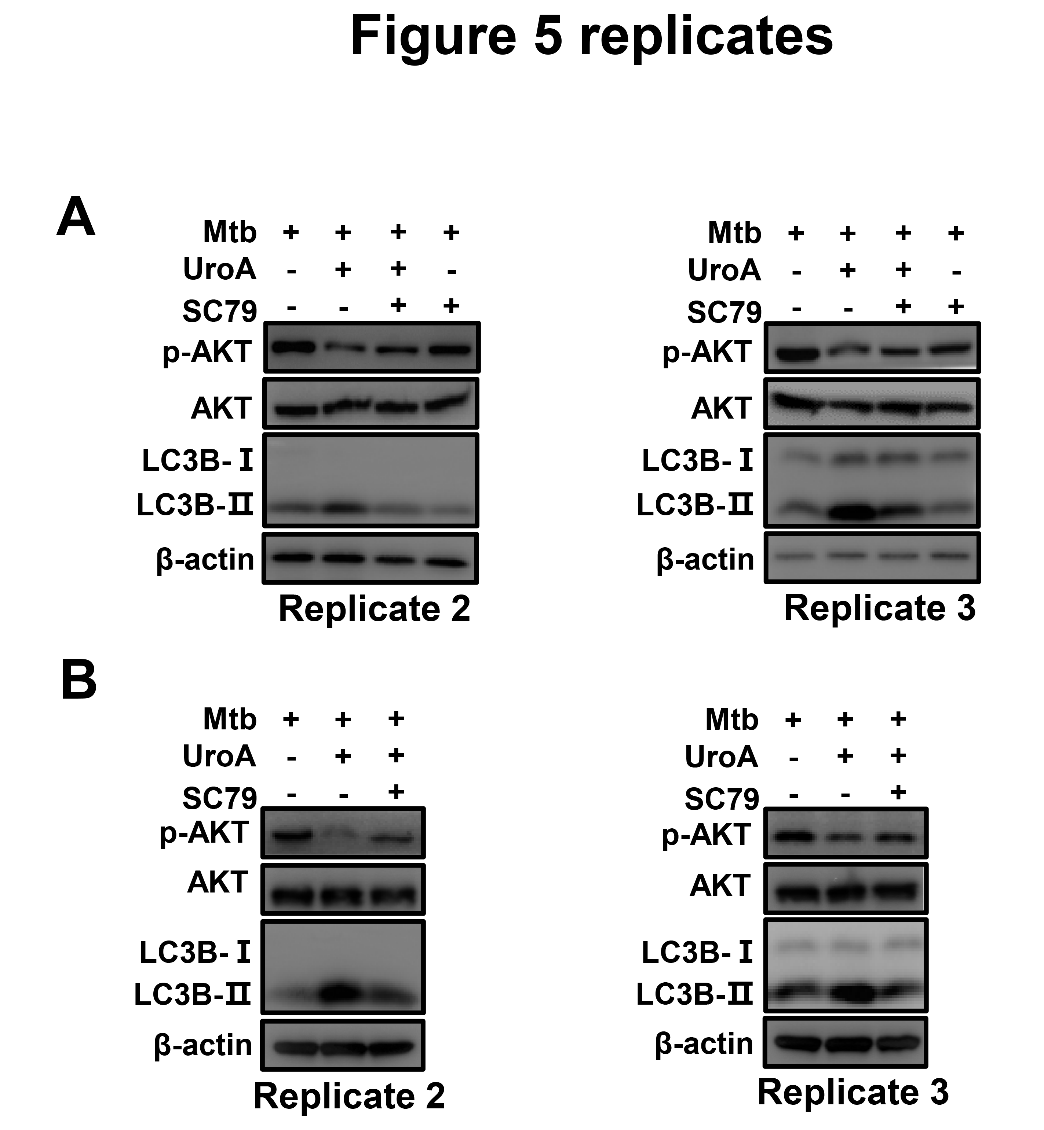


**Supplementary Figure 3. Western blot replicates for Figure 5.** **(A)** Western blot replicates for Figure 5A. **(B)** Western blot replicates for Figure 5C.


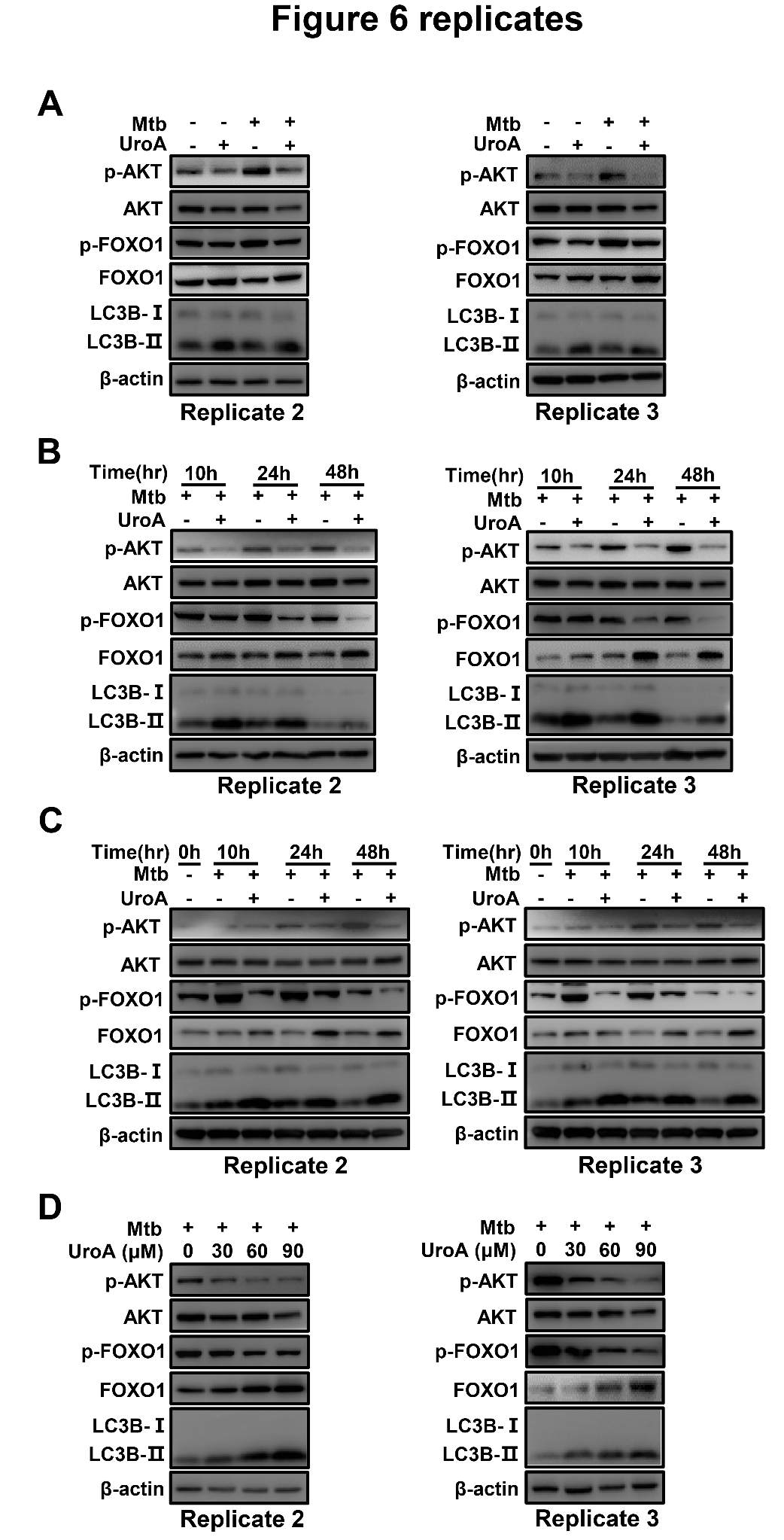


**Supplementary Figure 4. Western blot replicates for Figure 6.** **(A)** Western blot replicates for Figure 6C. **(B)** Western blot replicates for Figure 6E. **(C)** Western blot replicates for Figure 6G. **(D)** Western blot replicates for Figure 6I.


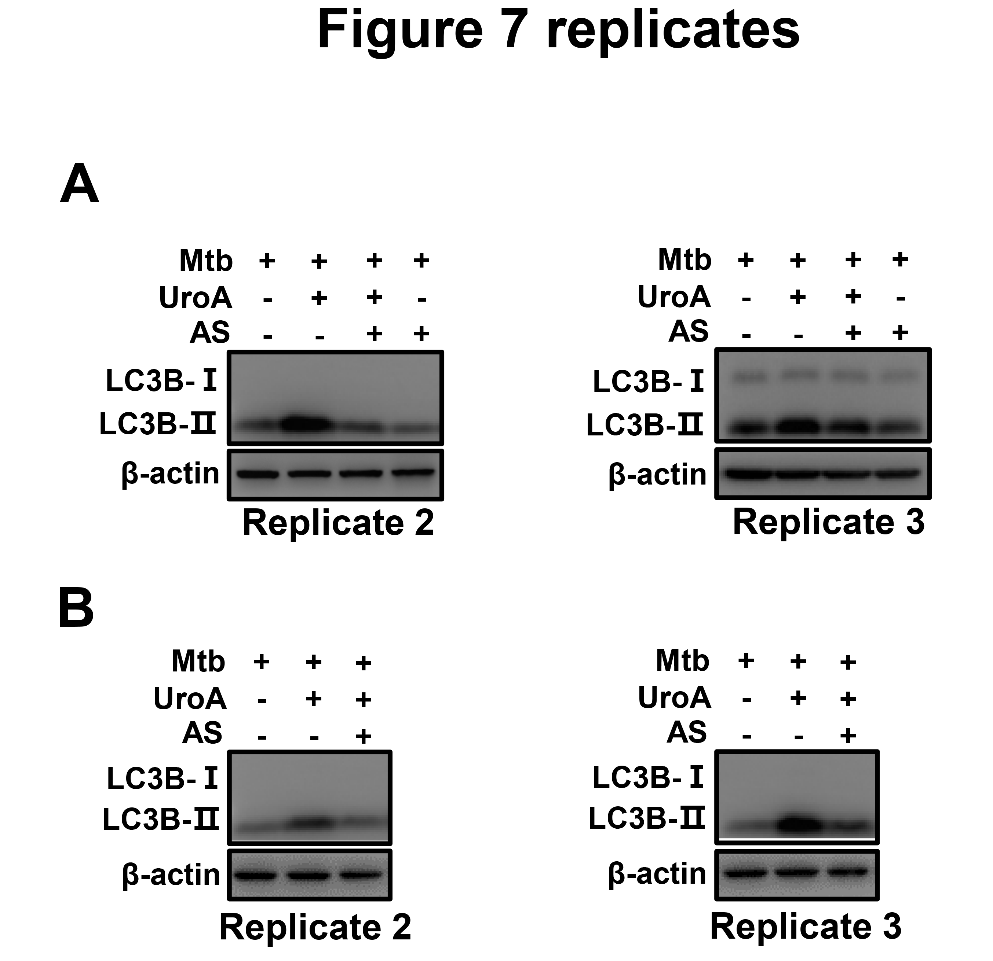


**Supplementary Figure 5. Western blot replicates for Figure 7.** **(A)** Western blot replicates for Figure 7A. **(B)** Western blot replicates for Figure 7C.
